# VIDEO RECORDING FOR CAPTURING INTERACTIONS BETWEEN RESIDENTS WITH DEMENTIA AND STAFF: A SYSTEMATIC REVIEW

**DOI:** 10.1093/geroni/igz038.1219

**Published:** 2019-11-08

**Authors:** Da Eun Kim, Hae Sagong, Eunjoo Kim, Ah Ram Jang, Ju Young Yoon

**Affiliations:** Seoul National University, Seoul, Korea, Republic of

## Abstract

The use of video-recording offers important advantages in observing and assessing the relationships between specific behaviors in health care settings. The purpose of this systematic review is to investigate and synthesize the methodological characteristics of studies using video-technology for measuring interactions between the older persons with dementia and staff in long-term care facilities. We searched Medline, Embase and CINHAL databases for published articles in English using a video-recording method for both staff and persons with dementia. Quantitative research design studies (e.g., descriptive studies or an experimental studies) were included. Among 5,605 searched papers, a total of 20 studies were selected for this review. Situations of video-recording were providing personal care (n=12), mealtime (n=6), and conversation (n=3). Concepts of measuring by video-recording were classified into two groups: 1) Staff [care practice by staff (n=13) and communication by staff (n=11)] and 2) residents [communication by resident (n=4), activities of daily life function of resident (n=8), and behavioral and psychological symptoms of dementia (n=10)]. This review demonstrates that video technologies are actively used to evaluate the relationship between quality of care and health outcomes of the elderly with dementia in many international nursing studies. This study provides the foundation for a future research using video-recording technologies to examine the interactions and relationship between staff and the residents in long-term care settings.

